# Industrial air pollution and lung and bronchus cancer survival in New Mexico, USA

**DOI:** 10.1007/s10552-026-02180-x

**Published:** 2026-05-26

**Authors:** Xi Gong, Yanhong Huang, Charles L. Wiggins, Angela L. W. Meisner, Yan Lin, Li Luo

**Affiliations:** 1 Department of Biobehavioral Health, The Pennsylvania State University, University Park, PA 16802 USA; 2 Institute for Computational and Data Sciences (ICDS), The Pennsylvania State University, University Park, PA 16802 USA; 3 Department of Geography & Environmental Studies, University of New Mexico, Albuquerque, New Mexico 87131 USA; 4 UNM Center for the Advancement of Spatial Informatics Research and Education (ASPIRE), University of New Mexico, Albuquerque, New Mexico 87131 USA; 5 University of New Mexico Comprehensive Cancer Center, Albuquerque, New Mexico 87131 USA; 6 New Mexico Tumor Registry, Albuquerque, New Mexico 87131 USA; 7 Department of Internal Medicine, University of New Mexico, Albuquerque, New Mexico 87131 USA; 8 Department of Geography, The Pennsylvania State University, University Park, PA 16802 USA; 9 Social Science Research Institute (SSRI), The Pennsylvania State University, University Park, PA 16802 USA

**Keywords:** Industrial air pollution, Lung and bronchus cancer, Survival, GIS, Environmental health, Exposure assessment

## Abstract

**Purpose:**

Lung and bronchus cancer (LBC) is the leading cause of cancer-related deaths. This study examines the relationship between exposure to industrial air pollution and lung and bronchus cancer survival (LBCS) in New Mexico, USA, from 1990 to 2019.

**Methods:**

This retrospective cohort study included data from 18, 273 lung and bronchus cancer patients. Residential exposure to nine industrial air pollutants was estimated for each patient using the Emission Weighted Proximity Model (EWPM), which integrates industrial air emission data and air quality monitoring data form the U.S. Environmental Protection Agency’s (EPA). Cox proportional hazards modeling was applied to identify industrial air pollutants as decreased LBCS risk factors, adjusting for age at diagnosis, gender, cancer stage, race/ethnicity, smoking prevalence, and urbanization of residential address.

**Results:**

Results show that residential exposure to 1,1,1-trichloroethane and cobalt during the survival period was significantly associated with decreased LBCS (adjusted hazard ratio [adjHR] for 1,1,1-trichloroethane: 1.06, 95% CI: 1.04, 1.09 and adjHR for cobalt: 1.06, 95% CI: 1.03, 1.09) compared to those unexposed among all patients. When exposure levels were categorized into four groups (unexposed, low, medium, high) based on annual average exposure during the survival period, the associations remained consistent.

**Conclusion:**

This study underscores the negative influence of industrial air pollution on LBCS and emphasizes the need for targeted public health interventions in high-exposure areas.

**Supplementary Information:**

The online version contains supplementary material available at 10.1007/s10552-026-02180-x.

## Introduction

Lung cancer is the leading cause of cancer-related deaths, accounting for 1.8 million deaths, or 18.7% of all cancer fatalities worldwide in 2022 [1]. It is also the third most commonly diagnosed cancer in the United States in 2024 [2]. The overall five-year survival rate for lung cancer in the United States was mere 27.5% during the period from 2014 to 2020 [3]. In New Mexico, lung and bronchus cancer (LBC) had an age-adjusted incidence rate of 32.7 per 100,000 population between 2017 and 2021, ranking as the second most commonly diagnosed cancer in the state [4]. Among racial and ethnic groups, the Asian/Pacific Islander (API) population had the highest incidence rate at 48.1 per 100,000 [5]. From 2018 to 2022, the age-adjusted mortality rate for LBC in New Mexico was 21.4 per 100,000, making it the leading cause of cancer-related mortality in New Mexico [4].

Lung and bronchus cancer survival (LBCS) is influenced by a range of factors, including age at diagnosis, sex, cancer stage at diagnosis, and comorbidities such as cardiovascular, hypertension, and diabetes [6–10]. Increasing evidence links air pollution, particularly fine particulate matter (PM), nitrogen dioxide (NO_2_), ozone (O_3_), sulfur dioxide (SO_2_), and other pollutants, to reduce lung cancer survival [11–13]. Globally, air pollution was estimated to contribute approximately 14.1% to lung cancer deaths, making it the second leading cause after tobacco smoking in 2017 [14]. Studies in specific regions further illustrate the associations between air pollution and lung cancer survival. In northern China, significant correlations were observed between long-term exposures to PM_10_ and SO_2_ and decreased lung cancer survival from 1988 to 2009 [15]. In the Californian, United States, air pollution exposures to NO_2_, O_3_, PM_10_, PM_2.5_ after diagnosis for lung cancer patients can significantly shorten survival from 1988 to 2009 [16]. Additionally, a strong association was observed between elevated blood lead (Pb) levels and decreased lung cancer survival among lead-exposed workers from 2003 to 2004 [17]. Similarly, in South Korea, exposure to pollutants such as SO_2_, carbon monoxide (CO), NO_2_, PM_2.5_, and PM_10_ was linked to reduced 1-, 3-, and 5-year lung cancer survival rates from 2009 to 2022 [18]. Furthermore, residential exposure to PM_2.5_, NO_2_, and black carbon (BC) was positively associated with reduced lung cancer survival across Denmark, England, Norway, and Rome (Italy) based on the corresponding cohorts during 2000 to 2017 [19]. Together, these findings highlight the urgent need to reduce air pollution as a key component of global efforts to increase lung cancer survival.

The most commonly studied air pollutants linked to decreased lung cancer survival include PM, O_3_, SO_2_, CO, Pb, and NO_2_, which are the criteria air pollutants (CAPs). Industrial air pollution is a significant contributor to environmental contamination, which is generated by factories, power plants, refineries, and various other industrial facilities [20, 21]. Common pollutants from these sources include PM, SO_2_, nitrogen oxides (NOₓ), volatile organic compounds (VOCs), and heavy metals, all of which pose serious health risks [22, 23]. Exposure to industrial air pollution has been associated with numerous adverse health outcomes, including respiratory and cardiovascular diseases, adverse birth outcomes, premature mortality, lung and breast cancer incidence, lymphoma, and hepatocellular carcinoma [24–28]. In addition, outdoor air pollution is a main contributor to lung cancer incidence globally [12]. These findings underscore the widespread associations of industrial air pollution on public health and suggest a potential link to lung cancer survival. However, the impact of many industrial air pollutants other than CAPs on lung cancer survival remains largely unexplored.

Despite known health risks of industrial air pollution, a research gap exists in understanding its specific link to LBCS, especially beyond commonly studied CAPs. The gap is particularly concerning for New Mexico, a state with a high incidence rate of lung and bronchus cancer, a longstanding mining legacy that has contributed to persistent environmental contamination, a largely rural landscape that complicates exposure assessment, ongoing socioeconomic challenges, and a racially and ethnically diverse population that is often underrepresented in environmental health research. These intersecting factors may contribute to disproportionate exposure burdens and poorer health outcomes, including lower cancer survival. Therefore, this study aims to explore the relationship between industrial air pollution and LBCS in the state of New Mexico from 1990 to 2019, with the potential to identify modifiable environmental risk factors and inform the development of targeted interventions and policies.

## Data and methods

### Lung and bronchus cancer data

As a comprehensive, population-based cancer registry for New Mexico, the New Mexico Tumor Registry (NMTR) provides high-quality cancer surveillance data to support scientific research and cancer control initiatives [29]. Our study cohort includes incident cases of LBC that were diagnosed among New Mexico residents during the time period 1990 to 2019. The NMTR collects all the LBC cases and individual-level data on various demographic and clinical characteristics from medical records, including age at diagnosis, sex, race/ethnicity, date of diagnosis, cancer stage at diagnosis, survival time (months), cause of death, date of last information (i.e., date of death for deceased patients and date last known to be alive for patients who have not yet died), and residential address at the time of diagnosis.

This study initially identified a cohort of 26,337 patients diagnosed with LBC in New Mexico between 1990 and 2019 using data from NMTR. We excluded cases based on the following: diagnosis reported only through autopsy or death certificate (5.87%), lack of microscopic confirmation with diagnostic sources such as laboratory, visualization, radiographic, clinical, or unknown (15.75%), missing geocoded residential coordinates (2.2%), missing or unknown cause of death (0.9%), missing survival months (6.2%), and missing cancer stage at diagnosis (3.1%) [30, 31]. After applying these exclusions, a final sample of 18,273 LBC cases remained for analysis. Among these cases, the median follow-up was 27 months, with 5.8% loss to follow-up likely due to incomplete linkage from missing identifiers.

Cancer site and morphology were coded according to the *International Classification of Diseases for Oncology*, Third Edition (*ICD-O-3)* [30]. According to The Surveillance, Epidemiology, and End Results (SEER) Program, cause-specific death classification variable is defined by taking into account cause of death in conjunction with sequence of tumor occurrence (i.e., only one tumor or the first of multiple tumors), site of the original cancer diagnosis, and comorbidities (e.g., AIDS and/or site-related diseases), with the aim of capturing deaths that were related to the specific cancer [32]. The SEER historic staging scheme provided information for in situ and invasive cancers, with the invasive cancers being divided into the following four stage categories: localized to the primary tumor site (localized), tumor with regional spread or metastases to regional lymph node (regional), tumor with distant metastases (distant), or unknown stage [30]. The diagnosis age (years) refers to the patient's age at the time of their first LBC diagnosis.

## Estimation of exposure

### Industrial air pollution emission and air quality monitoring data

**Fig. 1:**
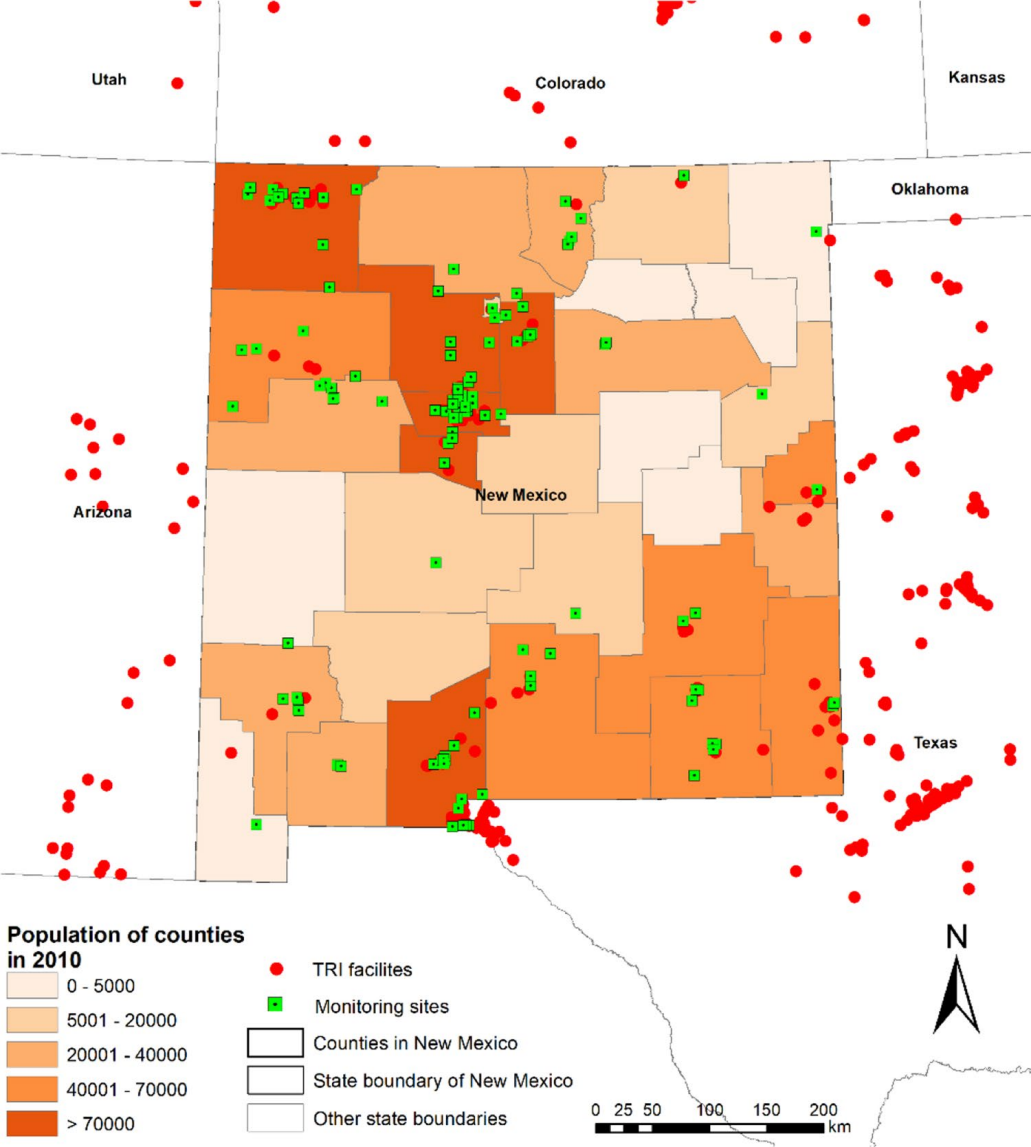
Geographic distribution of emission sources and monitoring sites in New Mexico and its surrounding areas during 1990–2019

The U.S. Environmental Protection Agency’s (EPA) Toxics Release Inventory (TRI) program is a federally mandated initiative requiring industrial facilities nationwide to submit annual reports detailing chemical emissions to the environment, including the types, quantities, and geographic locations of releases [33]. The primary objective of the TRI program is to monitor the management of toxic chemicals that pose potential risks to human health and the environment [33]. For this study, emission data were sourced from TRI records of industrial facilities located in New Mexico and neighboring areas, including northwestern Texas, southern Colorado, and eastern Arizona. Between 1990 and 2019, 577 industrial facilities across these regions released 188 distinct chemicals into the atmosphere (measured in pounds or grams per year) (Fig. 1), with annual emissions calculated by aggregating emissions from both stacks (emissions discharged through controlled exhaust systems) and fugitive sources (uncontrolled or diffuse emissions from equipment leaks, vents, and open processes). Additionally, air quality monitoring data were collected from the U.S. EPA’s Air Quality System (AQS) DataMart, which compiles data collected through the national ambient air monitoring program, including raw measurements and aggregated values (such as 8-h averages, daily averages, and annual averages) [34]. In New Mexico, between 1990 and 2019, 112 monitoring sites recorded data on 271 different air pollutants (measured in units of µg/m^3^, ng/m^3^, ppb, or ppm) (Fig. 1). To ensure consistency with the timescale of the emission data, annual average monitoring records were used for the subsequent analyses.

### Air pollution exposure assessment

This study utilized a calibrated Emission Weighted Proximity Model (EWPM) that was previously used to assess exposure to industrial air pollution at residential locations [35]. The EWPM estimates exposure intensity for a given pollutant at a given location by incorporating emission rate, duration, and distance from each emission source. Air pollutants from TRI are reported in units of pounds or grams per year, whereas chemicals from the AQS DataMart are measured in concentrations such as µg/m^3^, ng/m^3^, ppb, or ppm. The model has a key parameter, the effective distance, which defines the threshold beyond which emissions are excluded. Using monitoring sites as virtual exposure receptors, estimated exposure intensities at these locations were validated against monitoring data across varying effective distances. Therefore, effective distances were calibrated only for pollutants with both emission data and sufficient monitoring data (nine pollutants in this study), with each pollutant calibrated separately to minimize misclassifications of exposure assessment. The optimal effective distance for each chemical was determined by selecting the threshold that maximized the positive Spearman rank correlation between estimated and observed values. For further methodological details, please refer to [26, 35–38]. For each chemical (1,2,4-trimethylbenzene, chlorine, ethylbenzene, cobalt, chromium, manganese, mercury, copper, and 1,1,1-trichloroethane) with a calibrated optimal effective distance, we used the EWPM with the corresponding distance to estimate annual average exposure intensities at each case's residential location for every year throughout the survival period. The exposure period for each patient was defined as the time from the midpoint of the diagnosis year to the midpoint of the year of death or censoring.

### Identification of potential risk factors

We employed a Cox proportional hazards model (hereafter referred to as the Cox model) with time-dependent covariates to evaluate the associations (hazard ratios [HRs]) between residential exposure to industrial air pollution and lung and bronchus cancer survival (LBCS) in New Mexico from 1990 to 2019, adjusting for relevant covariates [39]. Annual average industrial air pollution exposure was defined as a time-dependent covariate to account for changes in exposure over the follow-up period. The selection of covariates in this analysis was guided by a directed acyclic graph (DAG), presented in Figure S1. Covariates included age at diagnosis, sex (male or female), race/ethnicity (Non-Hispanic (American Indian and Alaska Native) AIAN, non-Hispanic API, non-Hispanic Black, non-Hispanic Others, non-Hispanic White, and Hispanic), stage at diagnosis (localized, regional, distant, or unknown stage), and the urbanicity of the residential address (urban or rural). Race and ethnicity were combined into mutually exclusive categories by classifying all Hispanic individuals as Hispanic regardless of race and assigning non-Hispanic individuals to race-specific groups, ensuring each participant belonged to only one category. Urban and rural classifications were determined based on U.S. Census Bureau definitions, using census-designated criteria linked to the residential address at the time of diagnosis [40]. This study additionally adjusted for county-level current smoking prevalence (age 18 +) by incorporating available smoking data for cases diagnosed in corresponding years [41]. For observations with missing smoking data, we filled in the values using the average for that specific year. If data for a particular year were not available, we used the smoking rate from the closest year with available data. We assessed the proportional hazards assumption using Schoenfeld residuals and identified significant violations of the assumptions for two covariates: age at diagnosis and LBC diagnosis stage. To address these violations, we employed a Cox model across categories of age group (< 50, 50–59, 60–69, 70–79, and ≥ 80 years) and cancer stage (localized, regional, distant, and unknown), allowing the baseline hazard to vary by these factors. Each industrial air pollutant was evaluated individually in a single-pollutant Cox model, where it was included as the independent variable to assess its impact on LBCS. To examine potential effect modification by stage at diagnosis, we conducted stratified analyses using the Cox model separately for localized, regional, distant, and unknown stages, adjusting for all covariates except stage to capture stage-specific associations. In addition, to formally assess statistical evidence of effect modification, we fitted models that included an interaction term between pollutant exposure and stage at diagnosis.

The extended Kaplan–Meier estimator with time-varying covariates [42] was employed to estimate the overall survival information for the cohort, focusing on the key pollutants obtained in prior analyses (Sect. 2.2). Differences in survival distributions between exposed and unexposed groups were evaluated using the log-rank test. Median survival times and five-year survival probabilities were calculated for the entire cohort. To further examine the exposure–response relationships, patients in the exposed group were further categorized into low, medium, and high groups based on a cutoff at 0 exposure and the tertiles of annual average exposure intensities among those with exposures greater than 0 during the survival period. These exposure groups were compared to the unexposed group (exposure intensity = 0), respectively. Additionally, a Wald test for ordinal exposure levels was conducted to assess the significance of the trend of the associations. Bonferroni correction [43] was applied by dividing the nominal significance level (*α* = 0.05) by the number of comparisons (*k* = 45), and only associations with p-values below this adjusted threshold were considered statistically significant.

To explore potential interactions among the identified chemicals, we incorporated each pair of the nine chemicals into our two-pollutant Cox model, one pair at a time. For each pair, covariates were adjusted as outlined previously, ensuring that the variance inflation factor (VIF) for each variable in the model remained below 5 to avoid multicollinearity. In this study, individual air pollution exposure was assessed based on each participant's residential location. To account for uncertainties in potential exposure due to mobility beyond residential locations, we also conducted exposure assessments across different spatial scales, including census tract (2010) and zip code levels. Subsequently, individual-based exposure estimates were replaced with average exposure levels at the census tract and zip code levels corresponding to the residential location in the Cox models. This approach sought to provide a more comprehensive assessment of individual exposure by integrating spatial mobility factors. In addition, to assess local variation in the associations, we conducted county-specific Cox models across New Mexico, incorporating distance-based weights defined as the inverse of the distance between each individual’s residence and the centroid of the respective county.

## Results

The descriptive statistics and demographic characteristics of the study cohort are summarized in Table 1. The analysis included 18,273 patients diagnosed with LBC between 1990 and 2019. The cohort was primarily composed of males (54.99%) and non-Hispanic Whites (74.43%), with an average age of 69.50 years at diagnosis. Most patients were diagnosed at distant stage of LBC, comprising 52.86% of the cohort. Over the study period, 84.40% patients (*n* = 15,422) died specifically of LBC. Additionally, the majority of patients (77.94%) resided in urban areas at the time of diagnosis. The median survival time varied across cancer stages, with patients in the localized stage exhibiting median survival of 32 months, while those diagnosed at the distant stage had median survival of 4 months.

**Table 1:** Characteristics of patients with lung and bronchus cancer (LBC) in New Mexico by stage of diagnosis, summarized across all stages (All) and deaths, 1990—2019

Characteristics	LBC diagnosis							LBC death
	Localized (n = 2855)		Regional (n = 4290)	Distant (n = 9659)	Unknown (n = 1469)		All (n = 18,273)	Death (n = 15,422)
Race/ethnicity (n (%))								
Non-Hispanic White	2125 (74.43)	3186 (74.26)		6906 (71.50)	1001 (68.14)	13,218 (72.34)		11,198 (72.61)
Non-Hispanic Black	57 (1.89)	79 (1.84)		187 (1.94)	26 (1.77)	349 (1.91)		301 (1.95)
Hispanic	606 (21.23)	909 (21.19)		2294 (23.75)	398 (27.09)	4207 (23.02)		3532 (22.90)
American Indian and Alaska Native	46 (1.61)	78 (1.82)		176 (1.82)	23 (1.57)	323 (1.77)		274 (1.78)
Asian/Pacific Islander	11 (0.39)	34 (0.79)		79 (0.82)	9 (0.61)	133 (0.73)		101 (0.65)
Non-Hispanic Others	10 (0.35)	4 (0.09)		17 (0.18)	12 (0.82)	43 (0.24)		16 (0.10)
Sex (n (%))								
Male	1570 (54.99)	2519 (58.72)		5584 (57.81)	854 (58.13)	10,527 (57.61)		8916 (57.81)
Female	1285 (45.01)	1771 (41.28)		4075 (42.19)	615 (41.87)	7746 (42.39)		6506 (42.19)
Urbanicity (n (%))								
Urban	2282 (79.93)	3379 (79.52)		7481 (79.18)	1100 (74.88)	14,242 (77.94)		12,026 (77.80)
Rural	573 (20.07)	911 (20.48)		2178 (20.82)	369 (25.12)	4031 (22.06)		3396 (22.20)
Death (n (%))								
Alive/Death (other causes)	962 (33.70)	740 (17.25)		914 (9.46)	235 (16.00)	2851 (15.60)		-
Death (cancer cause-specific)	1893 (66.30)	3550 (82.75)		8745 (90.54)	1234 (84.00)	15,422 (84.40)		-
Age at diagnosis (mean ± SD)	71.82 ± 9.24	69.26 ± 9.81		68.46 ± 10.42	72.54 ± 10.44	69.50 ± 10.21		69.34 ± 10.29
< 50	46 (1.61)	125 (2.91)		391 (4.05)	33 (2.25)	595 (3.26)		533 (3.46)
50—59	234 (8.20)	585 (13.64)		1493 (15.46)	131 (8.92)	2443 (13.37)		2132 (13.82)
60—69	770 (26.97)	1377 (32.10)		3138 (32.49)	358 (24.37)	5643 (30.88)		4771 (30.94)
70—79	1211 (42.42)	1554 (36.22)		3222 (33.36)	555 (37.78)	6542 (35.80)		5435 (35.24)
> = 80	594 (20.81)	649 (15.13)		1415 (14.65)	392 (26.68)	3050 (16.69)		2551 (16.54)
Current smoking prevalence (county-level) (%)	15.78	15.84		19.61	18.15	18.69		20.62
Median survival months (interquartile range)	32 (120)	20 (62)		4 (19)	12 (52)	12 (30)		12 (26)

^*SD* standard deviation^

Following the calibration of effective distances for the selected nine chemicals, exposure intensities for the nine chemicals estimated using the EWPM exhibited statistically significant positive associations (*p* < 0.05) with the corresponding monitoring data (Table S1). The optimal effective distances of these nine chemicals ranged from 11 to 50 km, with correlation coefficients varying between 0.060 and 0.999 (Table S1). Multiple comparison corrections were not applied at this stage to allow as many chemicals as possible to be selected for downstream exposure assessment.

Table 2 presents the adjusted HR (adjHR) for the impact of exposure to nine industrial air pollutants on LBCS in New Mexico, stratified by cancer stage (localized, regional, distant, unknown) and summarized across all stages (represented as all stages hereafter). Significant associations with decreased LBCS were observed for exposure to 1,1,1-Trichloroethane (adjHR: 1.06, 95% CI: 1.04, 1.09) and cobalt (adjHR: 1.06, 95% CI: 1.03, 1.09) in the all stages. Specifically, exposure to 1,1,1-trichloroethane and cobalt were associated with 6% higher risk of dying from LBC, respectively, after adjusting for covariates (Table 2). This finding indicates that among LBC patients diagnosed at the distant stage, exposure to 1,1,1-trichloroethane during the survival period was associated with 14% higher risk of dying from LBC compared to unexposed patients. Similarly, cobalt exhibited significant association with reduced LBCS in the distant stage (adjHR: 1.12, 95% CI: 1.06, 1.19), with no significant associations observed in the other stages. All the associations are adjusted by Bonferroni correction for multiple comparisons.

**Table 2:** Adjusted HR (95% CI) for the association between industrial air pollution exposure and lung and bronchus cancer survival (LBCS) in New Mexico, 1990—2019, stratified by cancer stage (Localized, Regional, Distant, Unknown) and summarized across all stages (All)

	Adjusted HR^b^ (95% CIs)					*p*-value for interaction of stratified stage and air pollution exposure
Polluant (CAS number)^a^	Localized	Regional	Distant	Unknown	All^c^	
1,1,1-Trichloroethane (71,556)	0.90 (0.78, 1.02)	1.01 (0.90, 1.12)	1.14 (1.07, 1.21) *	0.88 (0.60, 1.10)	1.06 (1.04, 1.09) *	0.002*
Cobalt (7,440,484)	1.00 (0.95, 1.07)	0.95 (0.88, 1.04)	1.12 (1.06, 1.19) *	0.79 (0.55, 1.05)	1.06 (1.03, 1.09) *	0.004*
Chromium (7,440,473)	1.00 (0.93, 1.10)	0.98 (0.90, 1.08)	1.07 (1.00, 1.15)	0.89 (0.72, 1.09)	1.03 (1.00, 1.08)	0.054
Copper (7,440,508)	1.02 (0.91, 1.15)	0.96 (0.86, 1.07)	1.02 (0.94, 1.09)	0.80 (0.60, 1.02)	1.02 (0.98, 1.09)	0.352
Ethylbenzene (100,414)	0.96 (0.85, 1.08)	1.02 (0.91, 1.24)	0.92 (0.85, 1.00)	0.85 (0.68, 1.10)	1.00 (0.94, 1.06)	0.231
Chlorine (7,782,505)	0.97 (0.82, 1.16)	0.96 (0.86, 1.16)	1.05 (0.98, 1.13)	0.80 (0.64, 1.00)	1.00 (0.95, 1.07)	0.104
Manganese (7,439,965)	1.02 (0.87, 1.22)	1.00 (0.90, 1.10)	1.06 (0.93, 1.30)	0.90 (0.75, 1.08)	1.00 (0.94, 1.02)	0.208
1,2,4-Trimethylbenzene (95,636)	0.96 (0.78, 1.29)	1.00 (0.87, 1.14)	0.90 (0.81, 1.01)	0.80 (0.58, 1.05)	0.98 (0.91, 1.07)	0.277
Mercury (7,439,976)	1.12 (0.97, 1.71)	1.00 (0.91, 1.09)	0.91 (0.80, 1.03)	0.73 (0.47, 1.02)	0.96 (0.91, 1.02)	0.437

*Statistically significant after Bonferroni correction for multiple comparisons at level 0.05

^a^ A unique identification number assigned by Chemical Abstracts Service (CAS) to every chemical substance described in the open scientific literature; order in ascending *p*-values of the adjusted HR in all stages

^b^ Adjusted for diagnosis age, gender, race/ethnicity, current smoking prevalence, and urbanicity

^c^ Adjusted for diagnosis age, gender, race/ethnicity, diagnosis stage, current smoking prevalence, and urbanicity

**Fig. 2:**
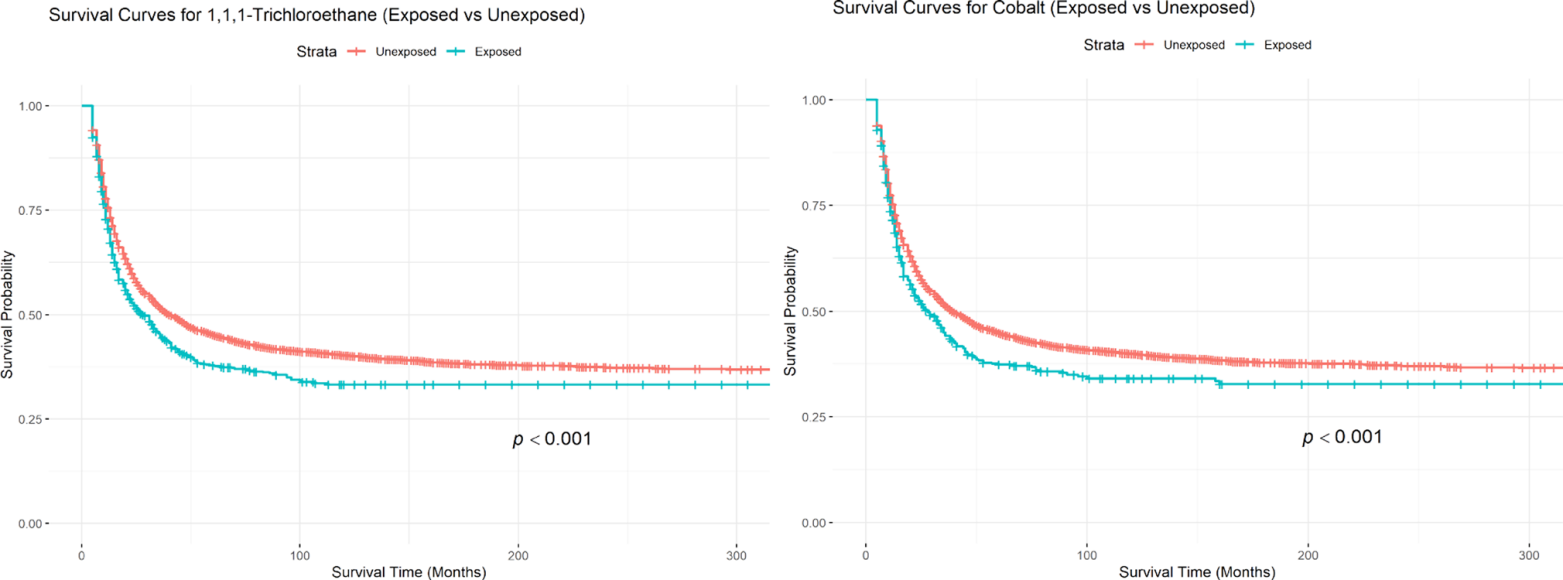
Kaplan–Meier Survival Curve for lung and bronchus cancer (LBC) patients of exposed and unexposed groups to 1,1,1-trichloroethane and cobalt

Table 3 presents the estimated median survival time and five-year survival rates for the cohort stratified by exposure to two significant air pollutants identified above (statistically significant adjHRs in the all stages in Table 2). For both pollutants, the unexposed group demonstrated a longer median survival time (15 months vs. 12 months) and higher five-year survival rates compared to the exposed group (1,1,1-trichloroethane: 27.11% vs. 21.49%; cobalt: 27.66% vs. 20.08%). Figure 2 depicts the survival curves for patients exposed and unexposed to the two air pollutants, illustrating the probability of survival over time (in months) for each group, and result of for Log-rank test (p-value in the figure). The unexposed group (represented in red) consistently exhibited a significant higher survival probability than the exposed group (represented in blue) throughout the studied period.

**Table 3:** Median overall survival time and five-year overall survival rate and air pollution exposure in New Mexico, 1990—2019

Pollutant (CAS number)^a^	Exposure intensity^b^	Median survival time in months (95% CIs)	Five-year survival rate in percentage (95% CIs)
1,1,1-Trichloroethane (71,556)	Unexposed	15 (14,15)	27.11 (26.02, 28.50)
1,1,1-Trichloroethane (71,556)	Exposed	12 (11,13)	21.49 (18.98, 24.84)
Cobalt (7,440,484)	Unexposed	14 (14,15)	27.66 (26.83, 29.04)
Cobalt (7,440,484)	Exposed	11 (10,13)	20.08 (18.25, 22.92)

^a^A unique identification number assigned by Chemical Abstracts Service (CAS) to every chemical substance described in the open scientific literature, order alphabetically

^b^Annual average residential air pollution exposure intensity during survival period

Table 4 shows the associations of varying levels of exposure (unexposed, low, median, high) to two pollutants identified as having significant adjusted HRs in the all stages on LBCS. For 1,1,1-trichloroethane, medium exposure was significantly associated with an increased risk of lung and bronchus cancer mortality, with a 7% (adjHR: 1.07, 95% CI: 1.03, 1.12) higher risk compared to the unexposed group. In contrast, associations for the low- and high-exposure groups were not statistically significant. For cobalt, high exposure was associated with a significant increased risk in dying from LBC (adjHR: 1.09, 95% CI: 1.04, 1.15), while both low and medium exposure levels showed no significant association with reduced LBCS. Notably, significant linear trends were observed among associations between residential exposure to both identified chemicals (1,1,1-trichloroethane and cobalt) and reduced LBCS, suggesting that higher exposure levels are associated with poorer survival..

**Table 4:** Adjusted HR (95% CI) for the association of exposure intensities of industrial air pollution and lung and bronchus cancer survival (LBCS) in New Mexico, 1990—2019

Pollutant (CAS number)^a^	Exposure intensity^b^	Mortality (LBC cause-specific)		Adjusted HR^c^	*p*-value for trend
		n	%		
1,1,1-Trichloroethane (71,556)	Unexposed (0)	16,258	90.27	1.00 (referenced)	< 0.001
1,1,1-Trichloroethane (71,556)	Low (0–2247.86)	434	2.41	0.95 (0.94, 1.13)	
1,1,1-Trichloroethane (71,556)	Medium (2247.87—6127.70)	523	2.90	1.07 (1.03, 1.12)*	
1,1,1-Trichloroethane (71,556)	High (> 6127.70)	805	4.47	1.08 (1.00, 1.17)	
Cobalt (7,440,484)	Unexposed (0)	16,874	93.64	1.00 (referenced)	< 0.001
Cobalt (7,440,484)	Low (0–35.99)	390	2.16	1.01 (0.93, 1.09)	
Cobalt (7,440,484)	Medium (36.00—68.21)	309	1.71	1.05 (0.99, 1.12)	
Cobalt (7,440,484)	High (> 68.21)	447	2.48	1.09 (1.04, 1.15)*	

*Statistically significant after Bonferroni correction for multiple comparisons at level 0.05

^a^ A unique identification number assigned by Chemical Abstracts Service (CAS) to every chemical substance described in the open scientific literature, order alphabetically

^b^ Annual average residential air pollution exposure intensity during the survival period

^c^ Adjusted for diagnosis age, gender, race/ethnicity, diagnosis stage, current smoking prevalence, and urbanicity

Compared to the Cox models with exposure to only a single pollutant, the adjHR showed slight variations when accounting for residential exposure to an additional pollutant (Fig.S4). Notably, 1,1,1-trichloroethane consistently demonstrated a significant positive association with decreased LBCS, even after adjusting for any of the other pollutants. In contrast, cobalt’s association became insignificant when adjusted for 1,1,1-trichloroethane (Fig.S4). These findings suggest that 1,1,1-trichloroethane may act as an independent risk factor for decreased LBCS, while cobalt may not. As shown in Table S2, results of the two identified chemicals at the census tract and zip code levels remained consistent with the individual-based estimates. For 1,1,1-trichloroethane, the adjHRs were 1.06 (95% CI: 1.04, 1.08) at the census tract level and 1.06 (95% CI: 1.03, 1.09) at the ZIP code level. Similarly, cobalt showed consistent associations across scales (census tract: HR, 1.06, 95% CI: 1.03, 1.09; ZIP code: HR, 1.05, 95% CI: 1.02, 1.09).

## Discussion

Our study contributes to the growing body of evidence linking air pollution exposure to LBCS. By analyzing nine industrial air pollutants across New Mexico from 1990 to 2019 using the large patient-level datasets collected by the NMTR, which is particularly significant given that LBC is the leading cause of cancer-related mortality in the state. The analysis employed a Cox model with time-varying covariates, enabling the modeling of dynamic exposure–response relationships over the survival period. We identified significant associations between residential air pollution exposure to 1,1,1-trichloroethane and cobalt and reduced LBCS in the all stages (all diagnose stages combined).

The findings are consistent with previous research indicating that exposure to air pollutants may exacerbate respiratory conditions and decreased cancer survival [44]. 1,1,1-trichloroethane (TCA), also known as methyl chloroform, is a volatile organic compound (VOC) that has been one of the most widely used cleaning and degreasing solvents in the United States [45, 46]. A study found that ambient concentrations of VOCs were associated with increased risk of cancer-specific death (HR: 1.06, 95% CI: 1.02, 1.11) in Toronto, Canada in 1982 to 2004 [47]. Exposure to certain metals is strongly associated with increased risk of lung cancer mortality due to their toxic, carcinogenic, and oxidative properties [48, 49]. For instance, elevated mortality rates from various cancers and respiratory diseases have been observed among members of the International Union of Bricklayers and Allied Craftworkers, potentially due to occupational exposure to cobalt and nickel [50]. In addition, studies have reported elevated lung cancer mortality among workers exposed to hexavalent chromium, with a standardized mortality ratio of 1.39 (95% CI: 1.17, 1.63) in Burbank, California, USA [51].

Fig. S2 shows average air pollution exposure intensities in New Mexico and nearby regions for the two identified chemicals during 1990–2019 estimated using the EWPM model. In New Mexico, emissions were mainly concentrated in the Albuquerque metropolitan area. Additionally, many industrial facilities in northern and western Texas (including the El Paso area) and eastern Arizona also released these chemicals into the air. Fig. S3 illustrates the local variation in adjusted HRs for the two identified chemicals, indicating that residents of Sandoval, Santa Fe, Los Alamos, Bernalillo, Valencia, and Torrance counties experience a higher risk of reduced LBCS associated with exposure to these chemicals. Future research may focus more specifically on these areas.

Based on the results presented in Table 4, a significant trend is observed in the adjHRs for increasing exposure intensities to 1,1,1-trichloroethane and cobalt. This suggests a positive association between higher exposure levels and higher risks of dying from LBC. Interestingly, residential exposure to low and high levels of 1,1,1-trichloroethane and low or medium levels of cobalt is not significantly associated with dying from LBC. This finding may be due to complex biological interactions, where low-level exposure may enhance antioxidant activity, while higher exposure levels likely surpass the body’s defensive capacity, resulting in oxidative stress, metabolic dysfunction, and an immunosuppressive environment that exacerbates cancer progression [52]. Further studies are essential to explore these potential explanations and to determine whether this association persists under specific conditions.

The consistency of the results across air pollution exposure assessments conducted at different spatial scales implies that the identified associations between air pollution exposure and LBCS are stable and robust to exposure estimation at finer or coarser spatial resolutions. The alignment of results across scales may indicate small variability in exposure levels within these larger geographic units or that the exposure contrast within residential neighborhoods is preserved across scales. Additionally, it reinforces confidence in the use of these alternative spatial aggregations, particularly when finer-scale data may be unavailable or when examining populations that may have spatial mobility.

To further assess the robustness of our findings, we conducted sensitivity analyses by incorporating block group level estimates of PM_2.5_ and NO_2_ as additional covariates, as well as by applying a 3-year lag of exposure prior. While PM_2.5_ and NO_2_ data were available for the same years as our primary dataset, they did not spatially align with the main industrial pollution exposures. Nevertheless, we included them separately in adjusted models to examine potential confounding effects. As shown in Table S3, the association with cobalt became statistically non-significant after Bonferroni correction when adjusted for PM_2.5_. The inclusion of a 3-year lag exposure yielded similar results, further supporting the stability of our findings.

This study also has a few limitations. First, individual-level variables such as dietary factors were not available in this analysis. These factors can influence the risk of decreased of LBCS and potentially introduce additional confounding but were not collected for the cohort during the data collection stage. Future studies should aim to collect more detailed individual-level data to better account for confounding factors and provide a more comprehensive understanding of the relationship between air pollution and lower LBCS risk.

Second, a proportion of cases (approximately 30%) were excluded due to data availability criteria. Although included and excluded cases were generally comparable in terms of demographic characteristics, urbanicity, and smoking prevalence, excluded cases included a higher proportion of older individuals (Table S4). This may result in a modest overestimation of survival time in analytic cases. While adjustment for age in the Cox models accounts for age differences within the analyzed dataset, it does not fully address potential selection bias due to the exclusion of older cases. The higher proportion of non-Hispanic White participants in this study which reflects the underlying demographic distribution of older adults in New Mexico, where a large portion of individuals aged > 50 years are non-Hispanic White. Additionally, smoking prevalence tends to be higher among non-Hispanic White populations compared to some other groups, which may further contribute to the observed distribution of lung cancer cases. The similar race/ethnicity distribution between included and excluded cases suggests that selection bias related to exclusion is likely limited, although generalizability to other populations should be considered.

Third, residential air pollution exposure was estimated based on residential locations which may not accurately reflect individual exposure levels. Variations in indoor pollution sources (e.g., radon exposure, dominant wind direction, and distance from industrial sources) and time spent on other places (e.g., 8 h a day or more in school or workplace) are not captured in this study, possibly leading to potential bias in the estimation of actual exposure. This limitation may result in exposure misclassification, which would likely bias estimated associations toward the null and attenuate observed associations. But the analysis conducted across different spatial scales indicates that the individual-based estimation is an appropriate scale for capturing residential air pollution exposure among patients. Additionally, this study did not explicitly adjust for time-varying environmental co-exposures, including O_3_ and CO, and meteorological factors such as prevailing wind direction and atmospheric dispersion. However, future studies could improve exposure assessment precision by incorporating mobility patterns, personal exposure monitoring, environmental co-exposures, and meteorological factors.

Fourth, our study used air monitoring data from New Mexico to calibrate the air pollution exposure model, where monitors are unevenly distributed across regions. This uneven distribution can introduce spatial bias, as monitoring sites are often concentrated in urban areas with higher population densities, leaving rural areas with limited coverage. Consequently, individuals residing in less monitored or rural regions may have less accurate exposure estimates. Future studies could address this limitation by incorporating advanced spatial interpolation techniques to enhance monitor coverage in under-monitored areas.

Finally, the spatial clustering of TRI emission sites may introduce spatial dependence in the exposure–outcome relationship that was not fully accounted for, and future analyses may benefit from addressing this issue.

## Conclusion

This study investigated the associations of industrial air pollution exposure on LBCS in New Mexico, USA, from 1990 to 2019. It is the first study in New Mexico to examine the relationship between air pollution and LBCS using individual-level data. The study identified significant associations between residential exposure to 1,1,1-trichloroethane and cobalt and a decreased LBCS. When exposure levels were categorized into four groups (unexposed, low, medium, high) based on annual average exposure during the survival period, the positive associations of the two air pollutants persisted. The extended Kaplan–Meier survival analysis indicated reduced survival probabilities for patients exposed to these two pollutants compared to those residing in unexposed areas during the study period. Additionally, chromium exposure has been found to be significantly associated with reduced LBCS among patients diagnosed at the distant stage. The findings underscore the urgent need for targeted public health interventions on reducing chemical exposures to improve patient survival outcomes. It is recommended that these findings be confirmed by further epidemiological, biological, and toxicological research.

## Supplementary Information

Below is the link to the electronic supplementary material.

Supplementary file1 (DOCX 1300 KB)

## Data Availability

The datasets generated during and/or analyzed during the current study are not publicly available due to Health Insurance Portability and Accountability Act.

## Abbreviations

adjHR: Adjusted hazard ratio
AIAN: American Indian and Alaska Native
API: Asian/Pacific Islander
AQS: U.S. EPA’s air quality system
BC: Black carbon
CAPs: Criteria air pollutants
CAS: Chemical abstracts service
CI: Confidence interval
CO: Carbon monoxide
Cox model: Cox proportional hazards model
DAG: Directed acyclic graph
EPA: U.S. Environmental protection agency
EWPM: Emission weighted proximity model
GIS: Geographic information system
HR: Hazard ratio
ICD-O-3: International classification of diseases for oncology, third edition
LBC: Lung and bronchus cancer
LBCS: Lung and bronchus cancer survival
NMTR: New Mexico tumor registry
NO_2_: Nitrogen dioxide
NOₓ: Nitrogen oxides
O₃: Ozone
PM: Particulate matter
SEER: Surveillance, epidemiology, and end results
SO_2_: Sulfur dioxide
TRI: U.S. EPA’s toxics release inventory
USA: The United States of America
VOC: Volatile organic compound
VOCs: Volatile organic compounds
