# Supplementary material for: Industrial air pollution and lung and bronchus cancer survival in New Mexico, USA
Source: Cancer Causes Control. 2026 May 26;37(6):98. doi: 10.1007/s10552-026-02180-x (PMC13212608; doi:10.1007/s10552-026-02180-x)
Supplement: Supplementary file 1 — Supplementary file1 (DOCX 1300 KB) [file 10552_2026_2180_MOESM1_ESM.docx]

**Supplemental Material**

**Industrial air pollution and lung and bronchus cancer survival in New Mexico, USA**

**Table of Contents**

Table S1 Calibrated parameters (effective distance k in EWPM) for selected chemicals.

Table S2 Adjusted HR (95% CI) for the association between industrial air pollution exposure and lung and bronchus cancer survival (LBCS) in New Mexico, 1990-2019, with exposure estimated across different spatial scales.

Table S3 Adjusted HR (95% CI) for the association between industrial air pollution exposure and lung and bronchus cancer survival (LBCS) in New Mexico, 1990-2019, with exposure estimated across different spatial scales.

Table S4 Characteristics of lung and bronchus cancer (LBC) patients in New Mexico, comparing included and excluded cases, 1990–2019.

Fig. S1 Directed acyclic graph (DAG) for the covariates in the Cox proportional hazards model with time-dependent covariates of this study, as supported by related literature.

Fig. S2 Average air pollution exposure intensity in New Mexico and its surrounding areas during 1990 - 2019 estimated using the EWPM model (a: 1,1,1-Trichloroethane, b: Cobalt).

Fig. S3 AdjHRs (95% CI) for the association between industrial air pollution exposure and lung and bronchus cancer survival (LBCS) across counties in New Mexico, 1990–2019 (a: 1,1,1-Trichloroethane, b: Cobalt).

Fig. S4 AdjHRs for single- and two-pollutant Cox models. The point reflects the adjHR, the vertical line represents the 95% confidence interval.

Table S1 Calibrated parameters (effective distance k in EWPM) for selected chemicals.

| Pollutant | CAS number^a^ | | | Spearman rank correlation test | | | | | |
| --- | --- | --- | --- | --- | --- | --- | --- | --- | --- |
|  | |  | | Optimal *k* (km) | | Coefficient^b^ | | *p*-value | |
| 1,2,4-Trimethylbenzene | | | 95636 | 11 | 0.999 | | < 0.001* | |  |
| Chlorine | | | 7782505 | 50 | 0.772 | | < 0.001* | |  |
| Ethylbenzene | | | 100414 | 11 | 0.999 | | < 0.001* | |  |
| Cobalt | | | 7440484 | 50 | 0.999 | | < 0.001* | |  |
| Chromium | | | 7440473 | 38 | 0.660 | | 0.001* | |  |
| Manganese | | | 7439965 | 50 | 0.491 | | 0.032* | |  |
| Mercury | | | 7439976 | 50 | 0.793 | | 0.033* | |  |
| Copper | | | 7440508 | 34 | 0.060 | | 0.039* | |  |
| 1,1,1-Trichloroethane | | | 71556 | 29 | 0.7 | | 0.047* | |  |

^a^ CAS Registry Number: A unique identifier assigned by the Chemical Abstracts Service to each substance; order in descending order of *p*-value.

^b^Sorted by ascending Spearman rank correlation *p*-value.

*Statistically significant (*p-*value < 0.05).

Table S2 Adjusted HR (95% CI) for the associations between industrial air pollution exposure and cancer-specific mortality due to lung and bronchus cancer (LBCS) in New Mexico, 1990-2019, with exposure estimated across different spatial scales.

|  | Adjusted^b^HR (95% CIs) | | | | |
| --- | --- | --- | --- | --- | --- |
| Polluant (CAS number)^a^ | Individual | Census tract | | Zip code | |
| 1,1,1-Trichloroethane (71556) | 1.06 (1.04, 1.09) * | | 1.06 (1.04, 1.08) * | | 1.06 (1.03,1.09) * |
| Cobalt (7440484) | 1.06 (1.03, 1.09) * | | 1.06 (1.03, 1.09) * | | 1.05 (1.02,1.09) * |
| Chromium (7440473) | 1.03 (1.00, 1.08) | | 1.03 (1.00, 1.07) | | 1.01 (0.98, 1.05) |
| Copper (7440508) | 1.02 (0.98, 1.09) | | 1.01 (0.99, 1.04) | | 1.00 (0.98, 1.03) |
| Ethylbenzene (100414) | 1.00 (0.94, 1.06) | | 0.99 (0.94, 1.04) | | 1.00 (0.95, 1.05) |
| Chlorine (7782505) | 1.00 (0.95, 1.07) | | 1.00 (0.96, 1.04) | | 1.01 (0.97, 1.05) |
| Manganese (7439965) | 1.00 (0.94, 1.02) | | 1.01 (0.94, 1.09) | | 0.99 (0.95, 1.04) |
| 1,2,4-Trimethylbenzene (95636) | 0.98 (0.91, 1.07) | | 0.97 (0.90, 1.06) | | 0.94 (0.90, 1.00) |
| Mercury (7439976) | 0.96 (0.91, 1.02) | | 0.94 (0.89, 1.01) | | 0.92 (0.87, 0.99) |

*Statistically significant following Bonferroni adjustment for multiple testing at the 0.05 level.

^a^ CAS Registry Number: A unique identifier assigned by the Chemical Abstracts Service to each substance.

^b^ Adjusted for diagnosis age, gender, race/ethnicity, diagnosis stage, current smoking prevalence, and urbanicity.

Table S3 Adjusted HR (95% CI) for the associations between industrial air pollution exposure and lung and bronchus cancer survival (LBCS) in New Mexico, 1990-2019; with additional adjustment for PM_2.5_ and NO_2_ separately, as well as sensitivity analyses incorporating a 3-year lag of exposure prior to diagnosis.

|  | Adjusted^b^HR (95% CIs) | | | | | | |
| --- | --- | --- | --- | --- | --- | --- | --- |
| Polluant (CAS number)^a^ | Model 1 | Model 2 | | Model 3 | | | Model 4 |
| 1,1,1-Trichloroethane (71556) | 1.06 (1.04, 1.09) * | | 1.05 (1.02, 1.09) * | | 1.05 (1.03, 1.08) * | 1.06 (1.03, 1.09) * | |
| Cobalt (7440484) | 1.06 (1.03, 1.09) * | | 1.06 (1.01, 1.11) | | 1.04 (1.01, 1.07) * | 1.05 (1.02, 1.09) * | |
| Chromium (7440473) | 1.03 (1.00, 1.08) | | 1.02 (0.95, 1.13) | | 1.05 (1.00, 1.10) | 1.03 (1.00, 1.10) | |
| Copper (7440508) | 1.02 (0.98, 1.09) | | 1.00 (0.93, 1.10) | | 1.00 (0.92, 1.09) | 1.02 (0.96, 1.09) | |
| Ethylbenzene (100414) | 1.00 (0.94, 1.06) | | 1.00 (0.90, 1.11) | | 1.00 (0.93, 1.08) | 1.00 (0.95, 1.06) | |
| Chlorine (7782505) | 1.00 (0.95, 1.07) | | 1.00 (0.94, 1.07) | | 1.00 (0.91, 1.09) | 0.98 (0.92, 1.05) | |
| Manganese (7439965) | 1.00 (0.94, 1.02) | | 0.94 (0.88, 1.02) | | 0.99 (0.81, 1.01) | 1.00 (0.92, 1.09) | |
| 1,2,4-Trimethylbenzene (95636) | 0.98 (0.91, 1.07) | | 0.96 (0.90, 1.03) | | 0.96 (0.90, 1.02) | 0.98 (0.92, 1.06) | |
| Mercury (7439976) | 0.96 (0.91, 1.02) | | 0.94 (0.82, 1.14) | | 0.90 (0.87, 1.02) | 0.92 (0.85, 1.01) | |

*Statistically significant after Bonferroni correction for multiple comparisons at level 0.05.

^a^ A unique identification number assigned by Chemical Abstracts Service (CAS) to every chemical substance described in the open scientific literature; order in ascending *p*-values of the adjusted HR in total stage.

Model 1 Adjusted for diagnosis age, gender, race/ethnicity, diagnosis stage, current smoking prevalence, and urbanicity.

Model 2 Adjusted for diagnosis age, gender, race/ethnicity, diagnosis stage, current smoking prevalence, urbanicity, and PM_2.5_.

Model 3 Adjusted for diagnosis age, gender, race/ethnicity, diagnosis stage, current smoking prevalence, urbanicity, and NO_2_.

Model 4 Three years lag effects: adjusted for diagnosis age, gender, race/ethnicity, diagnosis stage, current smoking prevalence, and urbanicity.

Table S4 Characteristics of lung and bronchus cancer (LBC) patients in New Mexico, comparing included and excluded cases, 1990–2019.

| Characteristics | Selected Cases (n=18,273) | Excluded Cases (n=8064) |
| --- | --- | --- |
| **Race/ethnicity (n (%))** |  |  |
| Non-Hispanic White | 13,218 (72.34) | 5840 (72.42) |
| Non-Hispanic Black | 349 (1.91) | 130 (1.61) |
| Hispanic | 4207 (23.02) | 1890 (23.43) |
| American Indian and Alaska Native | 323 (1.77) | 127 (1.57) |
| Asian/Pacific Islander | 133 (0.73) | 54 (0.70) |
| Non-Hispanic Others | 43 (0.24) | 28 (0.29) |
| **Sex (n (%))** |  |  |
| Male | 10,527 (57.61) | 4395 (54.50) |
| Female | 7746 (42.39) | 3669 (45.50) |
| **Urbanicity (n (%))** |  |  |
| Urban | 14,242 (77.94) | 6235 (77.32) |
| Rural | 4031 (22.06) | 1829 (22.68) |
| **Age at diagnosis (mean ± SD)** | 69.50 ±10.21 | 76.00 ±11.02 |
| <50 | 595 (3.26) | 159 (1.97) |
| 50-59 | 2443 (13.37) | 626 (7.76) |
| 60-69 | 5643 (30.88) | 1582 (1.96) |
| 70-79 | 6542 (35.80) | 2660 (32.97) |
| >=80 | 3050 (16.69) | 3037 (37.66) |
| **Diagnosis year (n (%))** |  |  |
| 1990-1994 | 2196 (12.02) | 859 (10.65) |
| 1995-1999 | 2678 (14.66) | 1034 (12.82) |
| 2000-2004 | 2979 (16.30) | 1540 (19.10) |
| 2005-2009 | 3117 (17.06) | 1864 (23.12) |
| 2010-2014 | 3582 (19.60) | 1470 (18.23) |
| 2015-2019 | 3721 (20.36) | 1297 (16.08) |
| **Current smoking prevalence (county-level) (%)** | 18.69 | 18.54 |


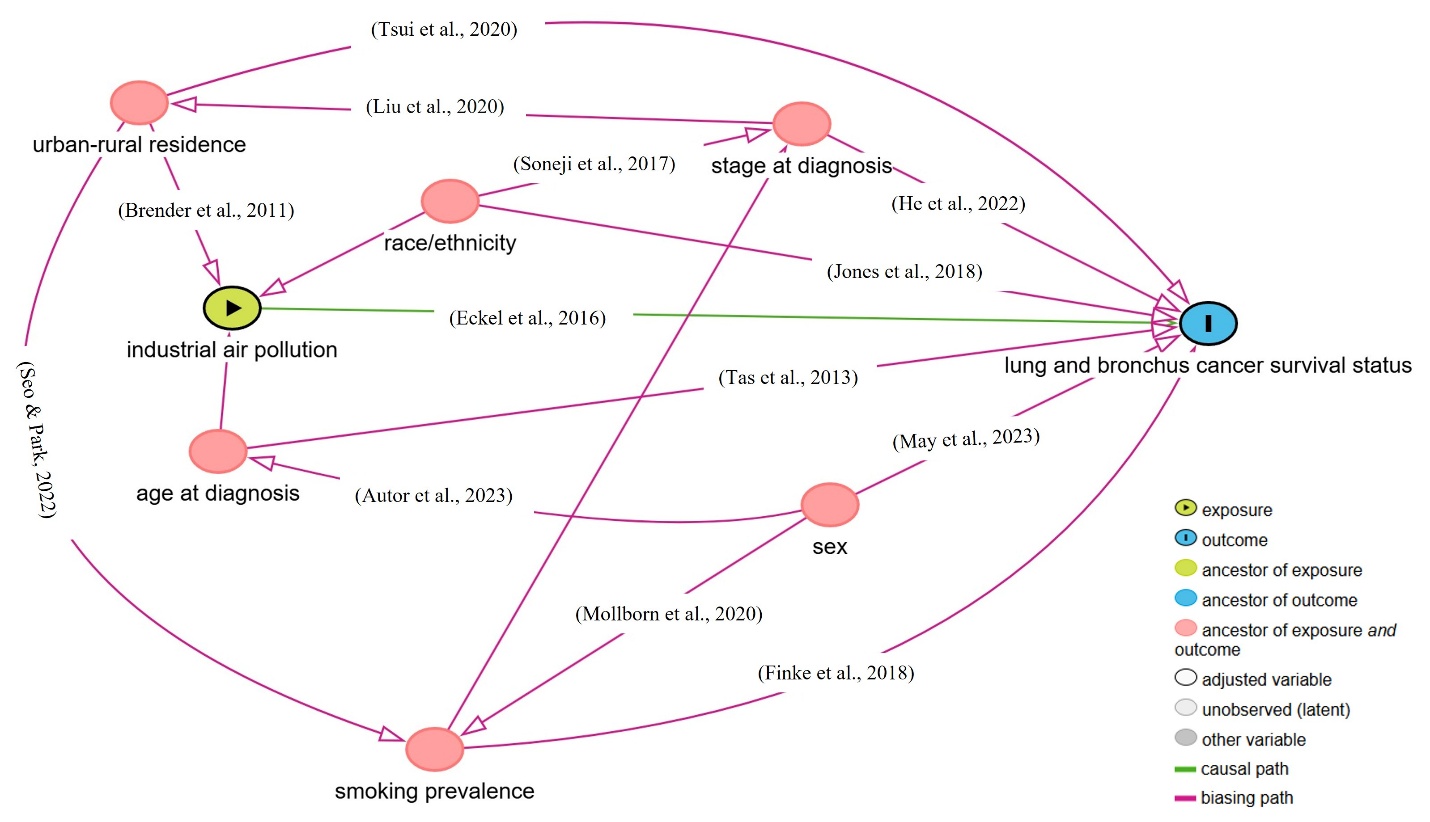


Fig.S1 Directed acyclic graph (DAG) for the covariates in the Cox proportional hazards model with time-dependent covariates of this study, as supported by related literature.


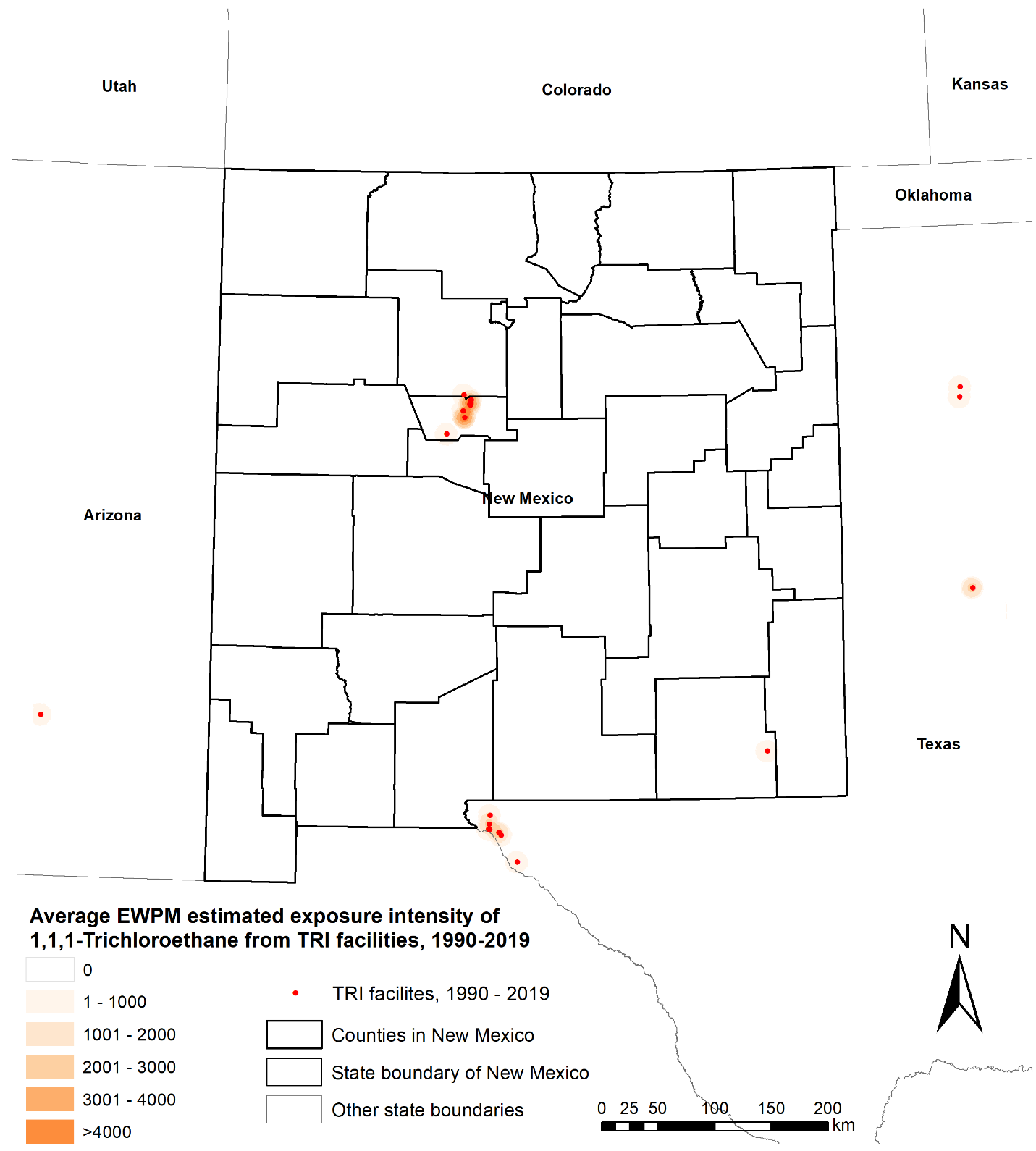


a


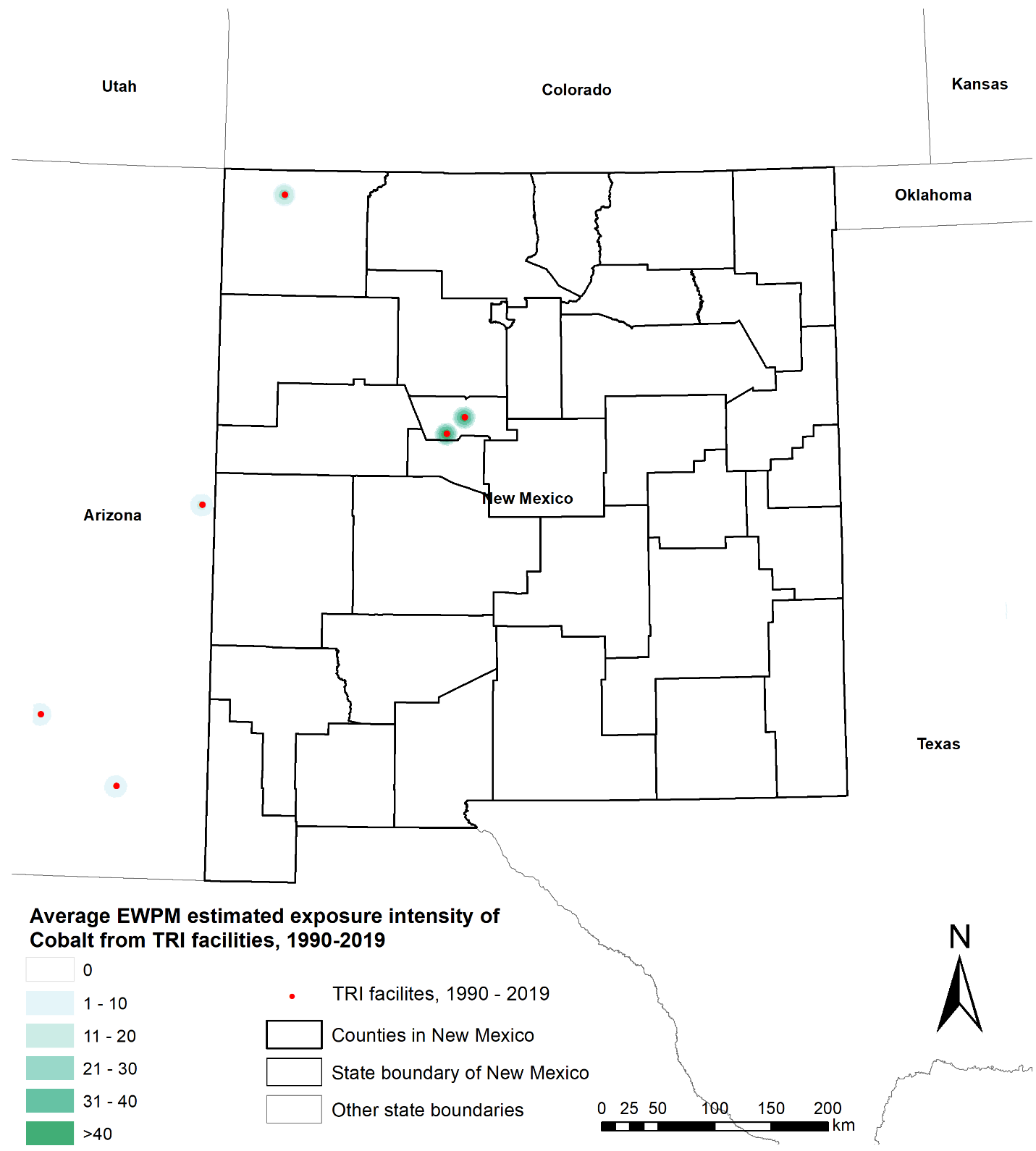


b

Fig.S2 Average air pollution exposure intensity in New Mexico and its surrounding areas during 1990 - 2019 estimated using the EWPM model (a: 1,1,1-Trichloroethane, b: Cobalt).


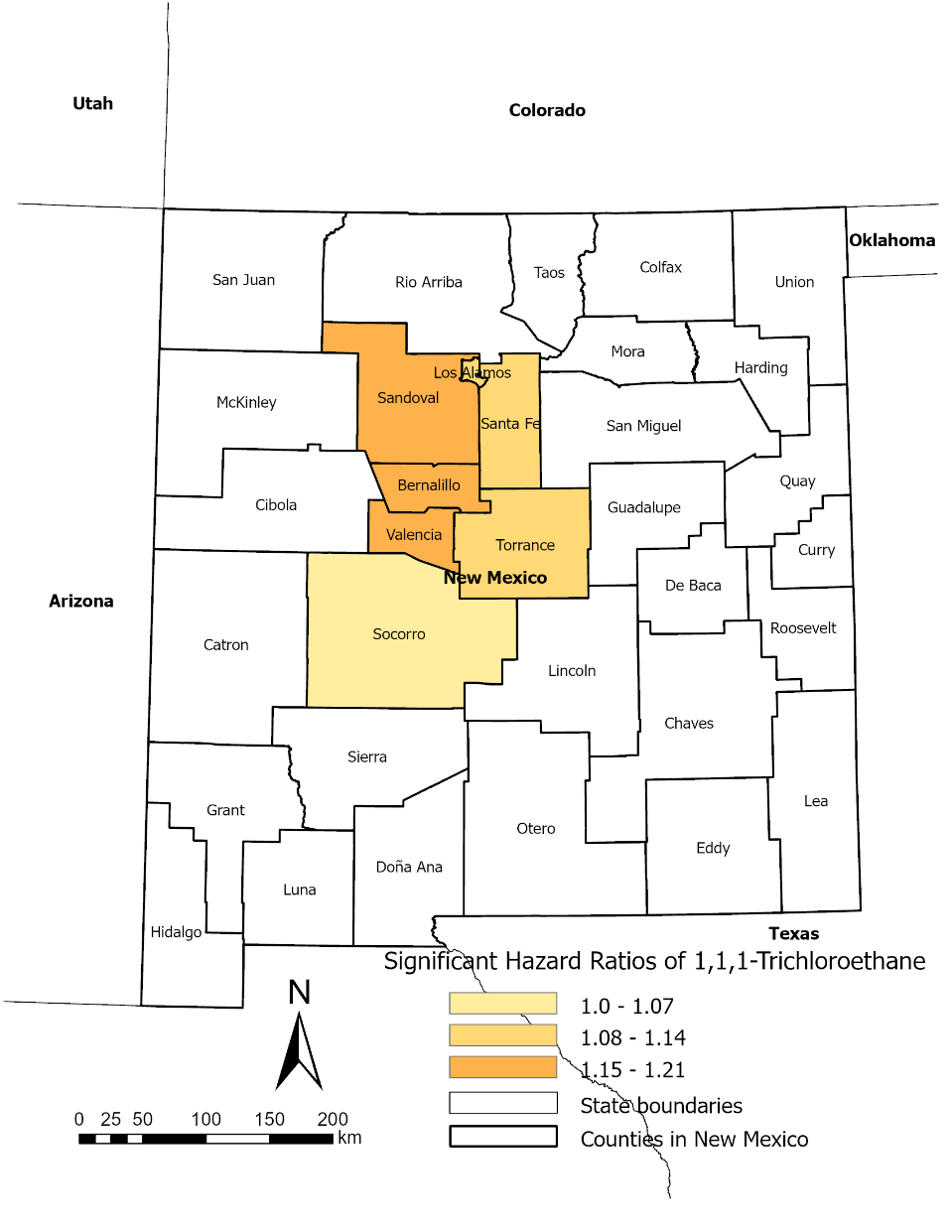


(a)


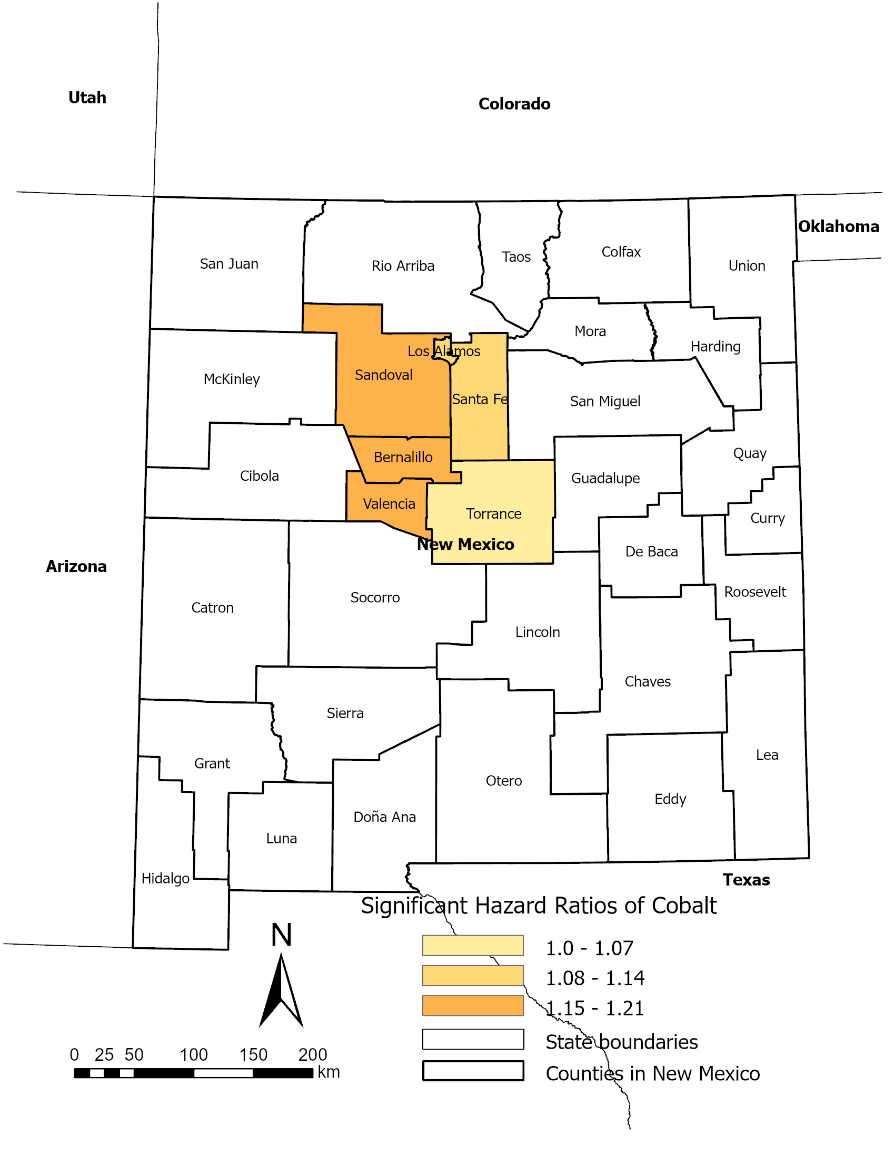


(b)

Fig. S3 AdjHRs (95% CI) for the association between industrial air pollution exposure and lung and bronchus cancer survival (LBCS) across counties in New Mexico, 1990–2019 (a: 1,1,1-Trichloroethane, b: Cobalt).


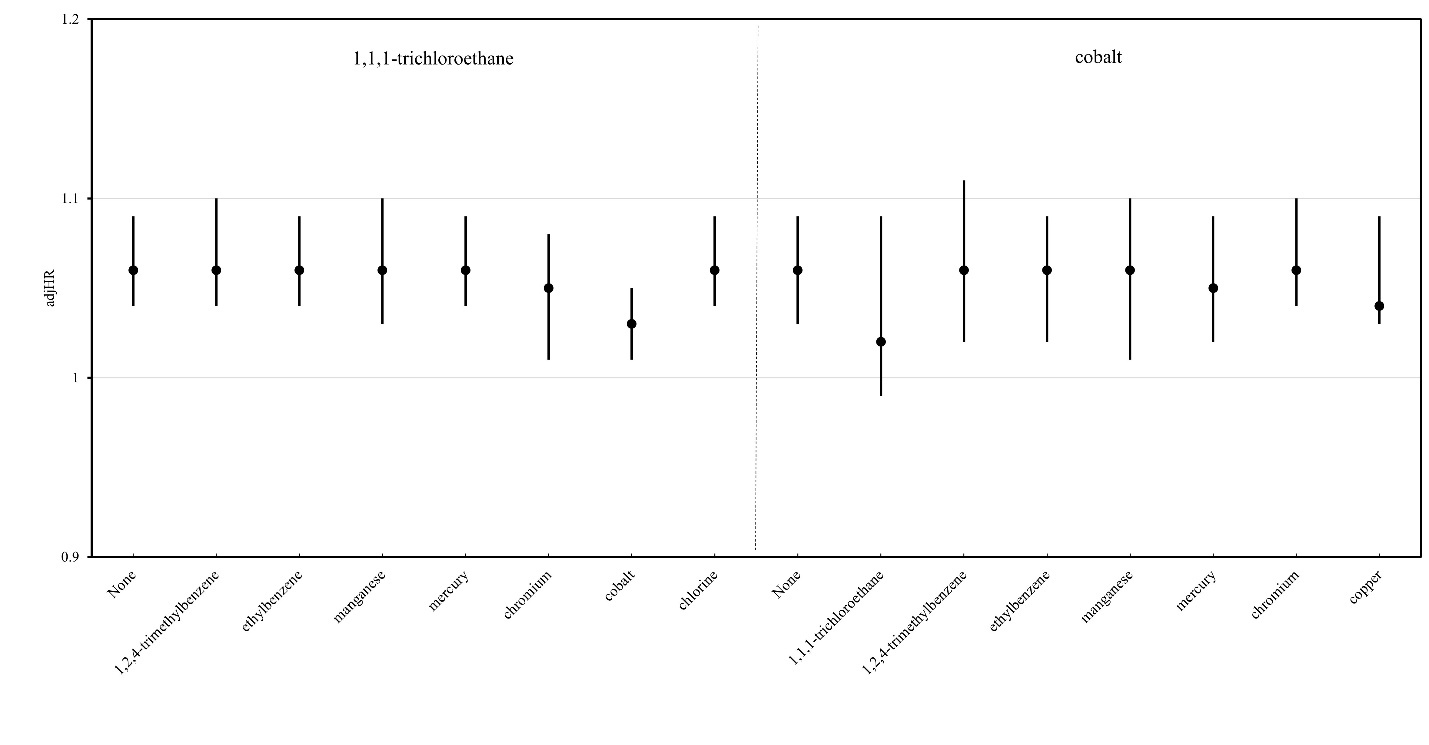


Fig.S4 AdjHRs for single- and two-pollutant Cox models. The point reflects the adjHR, the vertical line represents the 95% confidence interval.

**References**

Autor, D., Figlio, D., Karbownik, K., Roth, J., & Wasserman, M. (2023). Males at the Tails: How Socioeconomic Status Shapes the Gender Gap. *Economic Journal (London, England)*, *133*(656), 3136–3152. https://doi.org/10.1093/ej/uead069

Brender, J. D., Maantay, J. A., & Chakraborty, J. (2011). Residential proximity to environmental hazards and adverse health outcomes. *American Journal of Public Health*, *101 Suppl 1*(Suppl 1), S37-52. https://doi.org/10.2105/AJPH.2011.300183

Eckel, S. P., Cockburn, M., Shu, Y. H., Deng, H. Y., Lurmann, F. W., Liu, L. H., & Gilliland, F. D. (2016). Air pollution affects lung cancer survival. *THORAX*, *71*(10), 891–898. https://doi.org/10.1136/thoraxjnl-2015-207927

Finke, I., Behrens, G., Weisser, L., Brenner, H., & Jansen, L. (2018). Socioeconomic Differences and Lung Cancer Survival-Systematic Review and Meta-Analysis. *Frontiers in Oncology*, *8*, 536. https://doi.org/10.3389/fonc.2018.00536

He, S., Li, H., Cao, M., Sun, D., Yang, F., Yan, X., Zhang, S., He, Y., Du, L., Sun, X., Wang, N., Zhang, M., Wei, K., Lei, L., Xia, C., Peng, J., & Chen, W. (2022). Survival of 7,311 lung cancer patients by pathological stage and histological classification: a multicenter hospital-based study in China. *Translational Lung Cancer Research*, *11*(8), 1591–1605. https://doi.org/10.21037/tlcr-22-240

Jones, C. C., Mercaldo, S. F., Blume, J. D., Wenzlaff, A. S., Schwartz, A. G., Chen, H., Deppen, S. A., Bush, W. S., Crawford, D. C., Chanock, S. J., Blot, W. J., Grogan, E. L., & Aldrich, M. C. (2018). Racial Disparities in Lung Cancer Survival: The Contribution of Stage, Treatment, and Ancestry. *Journal of Thoracic Oncology : Official Publication of the International Association for the Study of Lung Cancer*, *13*(10), 1464–1473. <https://doi.org/10.1016/j.jtho.2018.05.032>

Liu, B., Lee, F. F., & Boscoe, F. (2020). Residential mobility among adult cancer survivors in the United States. BMC Public Health, 20(1). https://doi.org/10.1186/s12889-020-09686-2

May, L., Shows, K., Nana-Sinkam, P., Li, H., & Landry, J. W. (2023). Sex Differences in Lung Cancer. *Cancers*, *15*(12). https://doi.org/10.3390/cancers15123111

Mollborn, S., Lawrence, E. M., & Hummer, R. A. (2020). A gender framework for understanding health lifestyles. *Social Science & Medicine (1982)*, *265*, 113182. https://doi.org/10.1016/j.socscimed.2020.113182

Soneji, S., Tanner, N. T., Silvestri, G. A., Lathan, C. S., & Black, W. (2017). Racial and Ethnic Disparities in Early-Stage Lung Cancer Survival. *Chest*, *152*(3), 587–597. https://doi.org/10.1016/j.chest.2017.03.059

Tas, F., Ciftci, R., Kilic, L., & Karabulut, S. (2013). Age is a prognostic factor affecting survival in lung cancer patients. *Oncology Letters*, *6*(5), 1507–1513. https://doi.org/10.3892/ol.2013.1566

Tsui, J., Hirsch, J. A., Bayer, F. J., Quinn, J. W., Cahill, J., Siscovick, D., & Lovasi, G. S. (2020). Patterns in Geographic Access to Health Care Facilities Across Neighborhoods in the United States Based on Data From the National Establishment Time-Series Between 2000 and 2014. *JAMA Network Open*, *3*(5), e205105. https://doi.org/10.1001/jamanetworkopen.2020.5105
